# Transforming the invisible into the visible: disparities in the access to health in LGBT+ older people

**DOI:** 10.1016/j.clinsp.2022.100149

**Published:** 2022-12-17

**Authors:** Milton Roberto Furst Crenitte, Leonardo Rabelo de Melo, Wilson Jacob-Filho, Thiago Junqueira Avelino-Silva

**Affiliations:** a Laboratório de Investigação Médica em Envelhecimento (LIM 66), Serviço de Geriatria, Hospital das Clínicas da Faculdade de Medicina, Universidade de São Paulo (HCFMUSP), São Paulo, SP, Brazil; b Faculdade de Medicina da Universidade de São Caetano do Sul, São Paulo SP, Brazil; c Faculdade Israelita de Ciências da Saúde “Albert Einstein”, Hospital Israelita Albert Einstein, São Paulo, SP, Brazil

**Keywords:** Healthcare disparities, Sexual and gender minorities, Delivery of health care, Health promotion, Healthy aging

## Abstract

**Objectives:**

To compare variables of access to healthcare between the LGBT+ population aged 50 and over and those non-LGBT+.

**Methods:**

A cross-sectional study was carried out in Brazil through a confidential online questionnaire. The use of the health system was characterized by the number of preventive tests performed and measured by the PCATool-Brasil scale (a 10-point scale in which higher scores were associated with better assistance in healthcare). The association between being LGBT+ and access to health was analyzed in Poisson regression models.

**Results:**

6693 participants (1332 LGBT+ and 5361 non-LGBT+) with a median age of 60 years were included. In the univariate analysis, it was observed not only lower scores on the PCATool scale (5.13 against 5.82, p < 0.001), but a greater proportion of individuals among those classified with the worst quintile of access to healthcare (< 4 points), 31% against 18% (p < 0.001). Being LGBT+ was an independent factor associated with worse access to health (PR = 2.5, 95% CI 2.04‒3.06). The rate of screening cancer, for breast, colon, and cervical cancer was also found to be lower in the LGBT+ population.

**Conclusion:**

Healthcare access and health service experiences were worse in the LGBT+ group than in their non-LGBT peers. Inclusive and effective healthcare public policies are essential to promote healthy aging for all.

## Highlights

• LGBT+ people can face several inequalities in access to healthcare.
• LGBT+ people performed fewer preventive exams than their non-LGBT+ peers.
• The worst access score was found in black LGBT+ over 50 years old.

## Introduction

Factors such as poverty, sexual oppression, racism, and social exclusion may be associated with greater health vulnerability, in a model of vulnerability that goes beyond the understanding of individual factors or behaviors. It is also known that social and programmatic vulnerabilities are central aspects of the determinants of access to healthcare,^1^ which is particularly important in older adults, as they constitute the population that most often needs care.^2^

Researchers have used this broader concept of vulnerability also in aging, such as the study carried out by Rodrigues and Neri^2^ with a representative sample of older persons in a big Brazilian city. After a cluster analysis they found, i.e., a higher prevalence of urinary incontinence, loss of appetite, falls, and cognitive and sleep disorders among the poorest older adults.

However, there are still several relevant factors that need to be studied in the context of vulnerabilities and access to healthcare. In general, little is known about whether basic individual characteristics, such as gender and sexual orientation, interfere with access to health in Brazil and consequently, confer greater vulnerability on those who grow old.

Despite the achievements obtained by Lesbian, Gay, Bisexual, and Transgender (LGBT+) people in recent years, they are still often stigmatized and marginalized as a group, even in healthcare environments.^3^ Moreover, previous studies have shown that many LGBT+ people report feeling discriminated against in healthcare facilities and that they often avoid revealing their sexuality to professional providers.^4^

Considering this, Elliot et al.^3^ analyzed data from more than two million people in England (including 27,497 gay, lesbian and bisexual people) and when comparing the experiences of users in primary healthcare facilities, they observed that people belonging to sexual minorities reported unfavorable experiences about their care more frequently, including lack of trust in their physicians, perception of poor or very poor communication by doctors and nurses, and general dissatisfaction with their healthcare.

Likewise, a survey carried out in Ireland^4^ with 144 LGBT+ older adults found that only 43% (n = 51) of the sample felt respected as an LGBT+ person by healthcare services providers. Inequalities in their access to healthcare and previous negative experiences were also reported by older LGBT+ adults in Israel,^5^ in Australia,^6^ and in the United States of America.^7^

An ensuing concern is that an LGBT+ person would resist seeking medical attention due to the fear of discrimination and lack of trust in the system.^8^ Therefore, even if they sought medical care in emergencies, the LGBT+ population would be at a higher risk of inadequate follow-up and adverse outcomes.^6^ For example, one of the main works that investigated the influence of sexual orientation on lifestyle and access to health in older people was published in 2012 by Fredriksen-Goldsen e col.^9^ They gathered data from 96.992 North American aged 50 or over and concluded that lesbian and bisexual women were less likely to have had a mammogram. Other observational data confirm these findings and suggest that sexual minority women are less likely to perform a cervical pap smear than their heterosexual counterparts.^10^

Therefore, it is predictable that LGBT+ people would have unique health risks and complications compared to the heterosexual cisgender population.^11^ Numerous studies support this conclusion, reporting higher rates of depression, suicide, suicidal ideation, abuse of psychoactive substances, obesity, hypertension, and diabetes in lesbian, gay and bisexual citizens.^9^ These findings led researchers to hypothesize that belonging to a sexual or gender minority may be associated with an unhealthier aging process. Regardless, it is likely that models based mainly on “heterosexual aging” are insufficient to understand the peculiarities of the aging experiences in those with non-hegemonic expressions of sexuality.^12^

Despite the coexistence of a private health plan and a free and universal health system, Brazil is still a very unequal country, especially for LGBT+ people. There are quantitative data on LGBT+ older adults, but some groups of Brazilian non-governmental organizations disclosed some information supporting the idea that transgender people are at greater risk of dying before growing old.^13^

Thus, the present study aimed to investigate access to health in LGBT+ older people and to compare the results with an analysis of a corresponding heterosexual cisgender population, in order to refute the null hypothesis of non-inequality of access to health among them in Brazil.

## Methods

### Study design and population

A cross-sectional study was carried out involving Brazilians aged 50 years or older. Although the lower limit for the definition of older adults in Brazil is 60 years old, the cut-off age in our study was chosen based on previous studies that assessed the aging LGBT+ health.^14^, ^15^, ^16^

Participants were invited to respond to an anonymous online survey developed and managed with REDCap resources^17^ between August 2019 and January 2020. The study was announced in several medical associations, patient organizations, neighborhood associations, day-care centers and non-governmental associations. It also circulated on social networks, including Instagram, Facebook, WhatsApp, and Youtube. Visitors were encouraged to spread the information in their social groups, following “snowball sampling” recruitment strategies.^18^ To reduce the risk of recruitment bias, the questionnaire was published under the most generic scope of the research: the investigation of sociodemographic aspects associated with healthcare access, whose questions could be answered by both LGBT+ and non-LGBT+ people.

### Data collection and outcomes

Brazilians aged 50 years and over who consented to participate were eligible to complete a comprehensive questionnaire. The study exclusion criteria were the submission of incomplete data by the participant. Participants were invited to respond with their own sociodemographic and clinical information such as gender, date of birth, city of residence, skin color, marital status, religion, years of schooling, income, self-rated health perception, multimorbidity, polypharmacy, physical activity, tobacco and alcohol consumption, functionality,^19^ frailty,^20^ falls and depression.^21^

The number of preventive exams performed by the participants (mammograms, cervical pap smears, and colorectal cancer screening) was characterized and previous experiences of discrimination and harassment were questioned. The use of health services was assessed by the accessibility and first contact access issues contained in the PCATool Brasil scale (Supplementary Material S1),^22^^,^^23^ which is scored according to a Likert-scale (1-Indicating “Definitely not”, 2-Indicating “Probably not”, 3-Indicating “Probably yes”, 4-Indicating “Definitely yes” and 9-Indicating “Not sure/cannot remember”). The total score was calculated by summing (with reverse coding whenever appropriate) the values, and the total score was converted to a 10-point scale (in which higher scores determine better qualities of care):

Our primary independent variables were gender (cisgender male; cisgender female; transgender male; transgender female; *travestis*^24^; non-binary; other) and sexual orientation (heterosexual; homosexual; bisexual; pansexual; asexual; other). For analyses purposes, the authors have created an additional variable merging non-LGBT+ (cisgender male; cisgender female; heterosexual) versus LGBT+ participants (transgender male; transgender female; *travestis*; non-binary; homosexual; bisexual; pansexual; asexual; other).

Our primary outcome was access to healthcare according to the PCATool-Brasil scale. In their validation study for the Portuguese version, 6.6 points could define places with high accessibility for the first contact in health.^23^ In Brazil, Rech et al.^25^ found a mean of 4.24 points in the analysis of this domain. Our study also used the lowest quintile of the score obtained to define the group with the worst access to healthcare. The secondary outcome was assessed by the number of preventive exams performed by the participants (mammograms, pap smear and colorectal cancer screening).

As there is no consensus on the best translation of the term *travesti* and its translation may sound pejorative, in the present study it was decided to keep its nomenclature in Portuguese. *Travesti* is a transfeminine person who identifies with a *travesti* gender identity, which has been marginalized throughout history. It is predominantly an identity construction from Brazil, but it can be also found in other Latin American and European countries.^24^

### Statistical analysis

Statistical analyses were performed using Stata SE 15 (Stata Corp, College Station, Tx). All statistical tests were two-tailed, and an alpha error of up to 5% was accepted.

Measures of central tendency and dispersion counts, and proportions were used. Bivariate analyses using contingency tables, Chi-Squared test or Fisher's exact test, Student's *t*-test, or Wilcoxon's rank-sum test were performed when appropriate, comparing non-LGBT+ participants with LGBT+ participants. The authors also studied multivariable logistic regression with Poisson regression models to explore the adjusted association between belonging to the LGBT+ group and the worst quintile of access to healthcare, and the strengths of association were reported in prevalence ratios.

Initially, the following adjustment covariates were selected: age (years); sex assigned at birth; skin color; marital status; education; income (minimum wage[s]); use of the public health system; macro-region of the country; polypharmacy; arterial hypertension; diabetes *mellitus*; cancer; chronic obstructive pulmonary disease; asthma; Coronary disease; cardiac insufficiency; arthritis; stroke; chronic kidney disease; HIV infection; depression; frailty. The authors created a causal diagram to identify the minimum adjustment scheme for the association between being LGBT+ and access to healthcare based on these variables. In another moment, the authors sought to understand the intersectionality of oppression between gender, sexual orientation, and skin color through a multivariable analysis model in which skin color was placed as an interaction factor rather than being added separately as an adjustment factor.

Nevertheless, the LGBT+ population is not uniformly affected by the same stress factors, and previous studies suggest that transgender are subject to worse socioeconomic indicators and discrimination.^26^ For these reasons, a sensitivity analysis was also performed restricting our primary independent variable to cisgender persons.

### Ethical aspects

The IRB-Institutional Review Board of the University of São Paulo Medical School (FMUSP) approved the study – (approval number: 3.492.814). The online survey required eligible patients to read, understand and accept a Consent Form to participate. The questionnaires were anonymous and de-identified. And the authors followed strong guidelines to report our results.

## Results

**Fig. 1:**
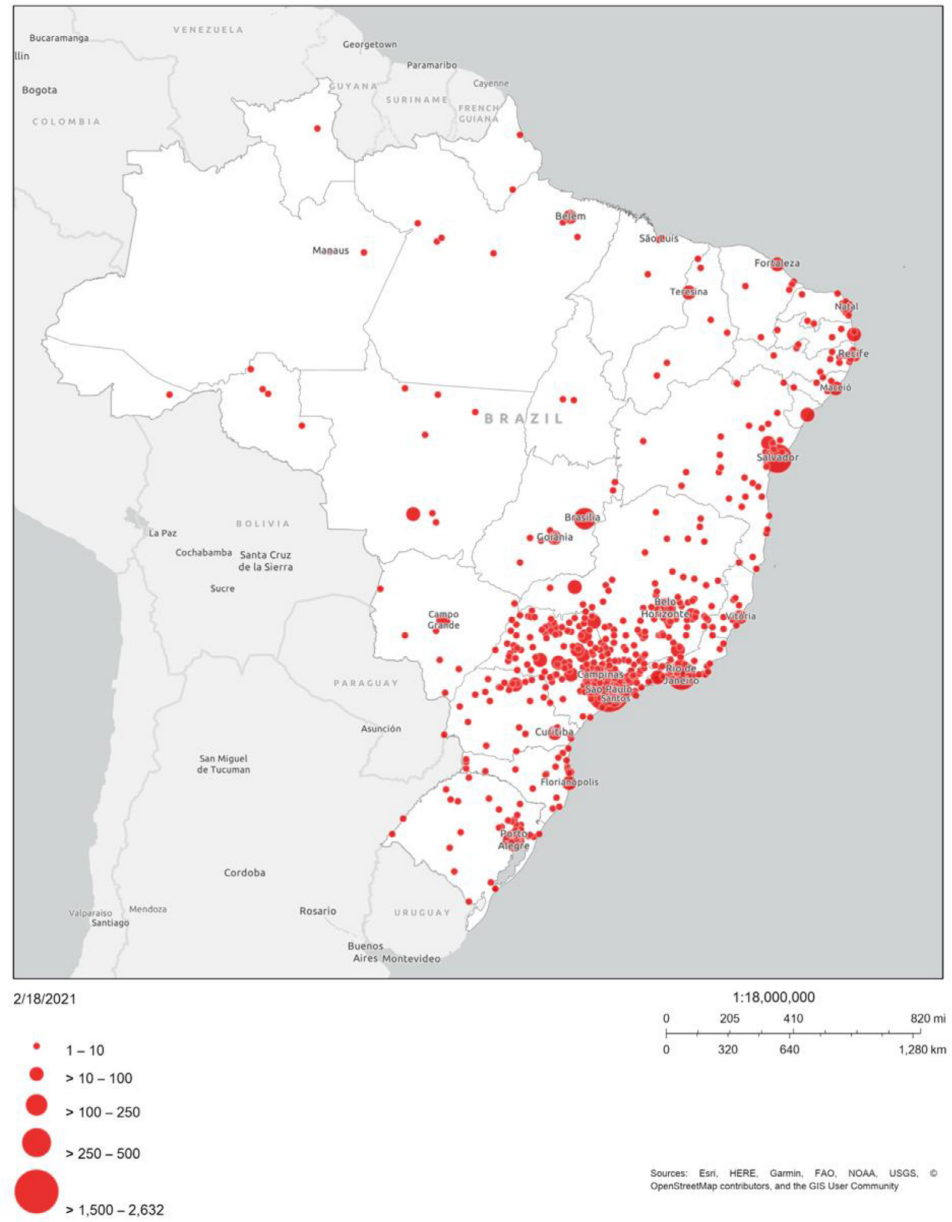
Geographic distribution of responses (n = 6,693). This figure represents the Brazilian territory. and the circles in red are related to the size of the sample from each highlighted city. Although all states of the Brazilian federation were represented. most responses were originated in the Southeast region. (For interpretation of the references to color in this figure legend, the reader is referred to the web version of this article.)

The median age of the population was 60 years, with an interquartile range of 55 to 66 years (Table 1) and a predominance of individuals aged 50‒59 years (65% in the LGBT+ group and 45% in the non-LGBT+ group, p < 0.001). Overall, 68% were female, with a predominance of this group being even more evident among non-LGBT+ participants (75% vs. 42%, p < 0.001). Most responses came from the Southeast region of Brazil (77%), as shown in Fig. 1, but all Brazilian states were represented. The authors managed to reach 6817 answers on the platform. However, 124 of them had incomplete data, therefore, were excluded. So, in the end, a total of 6693 participants were included in the final analysis.

**Table 1:** Baseline characteristics of the population, depending on whether they are LGBT+ or not (n = 6,693).

	Total	Non-LGBT	LGBT+	p-value
	(n = 6,693)	(n = 5,361)	(n = 1,332)	
Age (years), median (IQR)	60 (55, 66)	61 (55, 67)	57 (53, 62)	< 0.001
Age (years), n (%)				< 0.001
50‒59	3257 (49%)	2387 (45%)	870 (65%)	
60‒69	2490 (37%)	2112 (39%)	378 (28%)	
≥ 70	946 (14%)	862 (16%)	84 (6%)	
Sex assigned at birth, n (%)				< 0.001
Male	2115 (32%)	1343 (25%)	772 (58%)	
Female	4578 (68%)	4018 (75%)	560 (42%)	
Skin color or ethnicity, n (%)				< 0.001
White	5272 (79%)	4298 (80%)	974 (73%)	
Black	357 (5%)	301 (6%)	56 (4%)	
Others	1064 (16%)	762 (14%)	302 (23%)	
Marital Status, n (%)				< 0.001
Single	1051 (16%)	551 (10%)	500 (38%)	
Married	3749 (56%)	3206 (60%)	543 (41%)	
Divorced	1372 (20%)	1137 (21%)	235 (18%)	
Widower	521 (8%)	467 (9%)	54 (4%)	
Country Region, n (%)				< 0.001
Southwest	5186 (77%)	4241 (79%)	945 (71%)	
South	458 (7%)	350 (7%)	108 (8%)	
Central-West	247 (4%)	171 (3%)	76 (6%)	
Northeast	739 (11%)	553 (10%)	186 (14%)	
North	63 (1%)	46 (1%)	17 (1%)	
Religion, n (%)				< 0.001
Yes	5538 (83%)	4554 (85%)	984 (74%)	
No	991 (15%)	679 (13%)	312 (23%)	
Prefer not to answer	164 (2%)	128 (2%)	36 (3%)	

IQR, Interquartile range.

In the characterization of gender, most people identified themselves as cisgender (96%). Still, in the LGBT+ group, three people identified themselves as transgender men, 29 as transgender women, six as *travestis*, 68 as non-binary, and 100 as other gender identifications. Regarding sexual orientation in the LGBT+ group, 80 individuals recognized themselves as heterosexual, 939 as homosexual, 217 as bisexual, and 50 as pansexual.

While only 10% of heterosexual and cisgender people were single, this number reached 38% in the LGBT+ group (p < 0.001). The authors also found that most participants, regardless of being LGBT+ or not, had a high level of education, with 79% of them having attended higher education. Despite this, the authors found that LGBT+ people had a higher proportion of individuals with a low income (10% vs. 6%, p < 0.001) and were not homeowners (28% vs. 18%, p < 0.001).

Regarding screening tests, while 74% of heterosexual women reported having had at least one mammogram in their lifetime, only 40% of lesbians had the same report (p < 0.001). The number of LGBT+ people who underwent preventive screening for cervical or colon cancer was also lower, respectively 39% (against 73% among non-LGBT+ women, p < 0.001) and 50% (against 57%, p < 0.001). The doctor-patient relationship, an essential dimension for adequate access to healthcare, also has characteristics that draw attention among LGBT+ participants.

Among those who had a doctor and answered whether their doctors knew their gender identity or sexual orientation, 34% (n = 357) answered no. Of these, 40% said it would be important to talk about it with their physicians. Among LGBT+ participants who did not have a doctor, 51% (n = 69) responded that it would be important to have a healthcare professional in the field to talk about gender identity or sexual orientation. In contrast, 9% (n = 73) of LGBT+ participants who had a doctor and revealed their gender identity or sexual orientation perceived an inappropriate reaction from the professional. Overall, 53% of the LGBT+ group do not know or do not believe that physicians are prepared to handle the particularities of LGBT+ health.

The mean score of healthcare access by PCATool-Brasil was 5.68 (± 2.07), being 5.13 (± 2.02) in the LGBT+ group and 5.82 (±2.16) in the non-LGBT+ group (p < 0.001). The distributions of scores in the overall sample and subgroups are illustrated in Table 2. Regardless of the cut-off point used, the proportion of people with worse access to healthcare was higher in the LGBT+ group. Finally, the authors observed that the mean score for access to healthcare was particularly low among mixed-race or black LGBT+ people (4.62 [±2.05]) and black LGBT+ people (4.29 [±1.92]). Among these subgroups, respectively, 41% and 47% were in the worst access to health quintile.

**Table 2:** Healthcare access based on the Primary Care Assessment Tool (PCATool-Brasil), according to LGBT+ and skin color (n = 6,693).

	Total	Non-LGBT+ Not black or mixed	LGBT+ Not black or mixed	Non-LGBT+ black or mixed	LGBT+ Black or mixed	p-value
	(n = 6693)	(n = 4599)	(n = 762)	(n = 1030)	(n = 302)	
Mean score (SD)	5.68 (±2.07)	5.91 (±2.00)	5.25 (±2.05)	5.27 (±2.17)	4.62 (±2.05)	<0.001
Worst Healthcare access quintile (< 4 points), n (%)	1407 (21%)	775 (17%)	216 (29%)	291 (28%)	125 (41%)	<0.001
Pontuation < 4.24 points, n (%)	1654 (25%)	925 (20%)	247 (33%)	342 (33%)	140 (46%)	<0.001
Pontuation < 6.6 points, n (%)	4198 (63%)	2692 (59%)	529 (71%)	725 (70%)	252 (83%)	<0.001

SD, Standard Deviation.

As there are several individuals, social and programmatic factors potentially related to possible less access to healthcare, the authors used a multivariable regression model to investigate the association between being LGBT+ or not and accessing healthcare (Table 3). After elaborating a causal diagram, the following adjustment covariates were maintained in the model: age; sex designated at birth; skin color; marital status; education; income; use of the public healthcare system; habited country region; polypharmacy; arterial hypertension; diabetes mellitus; cancer; chronic obstructive pulmonary disease; Coronary disease; cardiac insufficiency; arthritis; stroke; chronic kidney disease; HIV infection; depression; frailty. The authors observed an independent association of the LGBT+ group with the worst healthcare access, maintaining all the cut-off points tested for scores by the PCATool-Brasil, with adjusted prevalence ratios ranging between 1.10 and 1.36. This finding was also observed in the sensitivity analysis performed, in which transgender people were excluded, with adjusted prevalence ratios of 1.34 (95% CI 1.17‒1.54) and 1.35 (95% CI 1.19‒1.53) for the worst healthcare access quintile and the national average, respectively. Furthermore, the authors did not verify interaction between skin color, LGBT+, and healthcare access.

**Table 3:** Poisson regression investigating the association between being LGBT+ and being in the worst quintile of healthcare access (PCATool-Brasil < 4.0) (n = 6,693).

	PR	95% CI		p-value	aPR	95% CI		p-value
LGBT+	1.66	1.48	1.86	<0.001	1.36	1.19	1.56	<0.001
Age	0.97	0.96	0.97	<0.001	0.98	0.97	0.98	<0.001
Sex at birth	1.02	0.91	1.14	0.716	1.12	0.98	1.27	0.098
Black skin color or mixed	1.70	1.50	1.92	<0.001	1.26	1.10	1.43	0.001
Marital status	Ref.				Ref.			
Single/ Divorced/ Widower	1.20	1.07	1.36	0.003	1.07	0.94	1.21	0.312
Single	1.55	1.35	1.78	<0.001	1.07	0.93	1.24	0.336
Education	Ref.				Ref.			
High school to incomplete college studies	1.45	1.28	1.64	<0.001	1.04	0.91	1.19	0.553
Midle school	1.59	1.24	2.04	<0.001	1.20	0.93	1.55	0.169
Income (US$)	Ref.				Ref.			
US$1000‒2000	2.14	1.86	2.46	<0.001	1.76	1.52	2.03	<0.001
< US$400	3.58	2.97	4.32	<0.001	2.13	1.73	2.63	<0.001
Public health utilization	2.51	2.24	2.80	<0.001	1.96	1.73	2.21	<0.001
Polypharmacy	0.79	0.70	0.89	<0.001	0.83	0.73	0.95	0.006
Arterial Hypertension	0.92	0.83	1.03	0.152	0.98	0.87	1.10	0.683
Diabetes	0.88	0.76	1.03	0.106	0.90	0.77	1.06	0.197
Cancer	0.61	0.46	0.82	0.001	0.71	0.53	0.95	0.022
COPD	0.98	0.74	1.31	0.908	0.91	0.67	1.24	0.559
Coronary Disease	0.96	0.72	1.29	0.799	0.99	0.72	1.37	0.961
Heart Failure	1.04	0.77	1.41	0.777	0.99	0.71	1.37	0.938
Arthritis	1.20	1.05	1.37	0.007	1.19	1.04	1.37	0.013
Stroke	0.97	0.62	1.50	0.882	0.86	0.55	1.35	0.521
Chronic Kidney Disease	1.41	0.96	2.08	0.083	1.29	0.87	1.93	0.205
HIV infection	1.76	1.42	2.17	<0.001	0.88	0.69	1.11	0.277
Depression	1.70	1.53	1.89	<0.001	1.32	1.18	1.48	<0.001
Frailty	1.78	1.49	2.13	<0.001	1.26	1.03	1.53	0.023

PR, Prevalent Ratio; Pra, Adjusted Prevalent Ratio; 95% CI, Confidence Interval 95%.

## Discussion

The authors hypothesized that older and middle-aged LGBT+ people would face several barriers when accessing healthcare services in Brazil, based on studies that report prejudice and other vulnerabilities they struggle with.^11^ Thus, in a cross-sectional study of more than 6,000 citizens aged 50 or more, the authors found that being LGBT+ was independently associated with more limited access to healthcare. We also observed that the worst scores by the PCATool-Brasil instrument occurred among black LGBT+ people and that cis lesbian women were less likely to undergo cancer screening tests than heterosexual cis women. The overall score of this instrument was higher than the Brazilian national average,^22^ which can be explained by our high number of participants with good socioeconomic conditions. However, black LGBT+ people in our sample scored lower than adults in Cape Town and Wineland (South Africa) districts.^27^ In relation to other international studies, the authors also found lower healthcare access scores than those found in Cordoba (Argentina),^28^ Seoul (South Korea)^29^ e and Quebec (Canada).^30^

Our study contributes to new evidence with observational studies that show worse health conditions and worse experiences in healthcare services by older LGBT+ people.^31^ Using the validated PCA-Tool, it was possible to objectively quantify these issues, including analysis of the interaction between LGBT+, skin color, and use of the public health care system. A 2018 publication with representative data on 1,263 older adults in Sao Paulo (Brazil)^32^ had already shown worse conditions of access to health for black older citizens, revealing in this group a higher proportion of those without private health insurance and fewer preventive exams. However, this research did not analyze the influence of belonging to a sexual and gender minority on these trends.

One of the main works investigating the influence of sexual orientation and access to healthcare in older people was published in 2013 by Fredriksen-Goldsen et al.^14^ They gathered data from 96,992 Americans aged 50 years and over, and despite showing that lesbian and bisexual women were less likely to undergo mammograms than heterosexuals, they concluded, after adjustments for sociodemographic variables, that access to healthcare was not inferior in sexual minorities. However, the study limited its assessment of healthcare access to three aspects: having a private health plan; having a reference health professional, or feeling a financial barrier to visiting a doctor in the past year. Our study deepens the analysis of access to healthcare for LGBT+ adults in a context of universal service and uses a more comprehensive and detailed questionnaire based on an objective measure of this question.

More recently, researchers have been working to identify potential barriers to this population's well-being. Among these recent efforts is the integrative revision conducted by Ferreira and Bonan,^33^ in which the authors classified the healthcare access of this community in three complex and interrelated dimensions: relational, organizational, and contextual. The first deals with the relationship between users and healthcare services providers, in which dignified reception and active listening are essential to ensure medical care of optimal quality. It may be pointed out as a condition to the revealing or not of the sexual orientation or gender identity by LGBT+ people in healthcare services, and also as an access barrier.^34^ It may be pointed out as a condition that helps to decide whether or not to reveal one's sexual orientation or gender identity in a healthcare environment. The obstacles of the organizational dimension are the cisgender heteronormative ways according to which services and processes are executed within the healthcare services, such as failure to recognize the social name of transgender people in communications, difficulties in accessing locations such as toilets, lack of educative material including LGBT+ people in waiting rooms and exposing them to shaming situations. Contextual barriers are social determinants of the health-illness process, such as poverty, violence, discrimination, and stigmatization, which widen the vulnerability of people and communities. LGBTphobia is one of these main determinants, especially when coupled with racism.

Despite being widely publicized for six months, only a few transgender people answered the survey. Therefore, it was not possible to gather representative data on this population, which is known for suffering high rates of violent death and suicide in Brazil. Researchers evaluate that, albeit in different situations, the transgender population is placed under analysis in the same group as lesbians, gays, and bisexuals, it is a group with distinct and heterogeneous characteristics. So, researching them can be challenging not only in recruiting participants but also in doing it with the best approach with the best questions. Therefore, future research on this topic must be thought and designed specially to study the reality of older transgender people.

### Strengths and limitations

The present study had some limitations. The main one refers to an evident bias in the selection of participants with better socioeconomic conditions than the average of the Brazilian population. As the survey was mainly disseminated on social media, requiring electronic resources for participation, the outreach to the most vulnerable communities was limited. The authors also highlight the low rate of responses from transgender people, limiting the present analysis of the reality experienced by this population group in our country. The use of an online questionnaire may also have limited the participation of older groups of participants. Even so, recent surveys showed an increase in their use of the internet in the last five years.

On the other hand, the present work also had important positive aspects. The authors were able to include a large number of participants, both non-LGBT+ and LGBT+, and to assess in detail various aspects of the health of therespondents. The anonymity of the participants is another central aspect of the investigation, as it increases the chance of getting truthful answers about subjects that are often considered taboo. Another strength of the work is the high rate of complete responses obtained, which corroborates the quality of the data found that supports the odds of good quality data. This is also a pioneering quantitative study in Brazil since, to date, most of the data used by Brazilian researchers to portray the profile of LGBT+ older people came from Anglo-Saxon contexts. It is also worth mentioning the use of PCATool-Brasil as a positive element in this study, as it is an objective instrument of healthcare measurement, validated for the Brazilian context. Recommended by the Ministry of Health for the evaluation of Primary Health Care, it is easy to use and allows comparison with other national and international publications.

## Conclusion

Healthcare access, the number of preventive tests performed, and experiences with health services were worse in the LGBT+ group than in their non-LGBT peers. Black skin color or brown/mixed race and use of the public health system were not confirmed as modifiers of the association between being LGBT+ and worse healthcare access, but both variables had an independent association with being in the worst quintile of healthcare access in the population. Public policies to reduce programmatic vulnerabilities are essential to include people from different backgrounds and profiles in health services. This inclusion process is essential to ensure that LGBT+ people be healthier and age better and to overcome the inequalities and the invisibility they face throughout their lives.

## Authors’ contributions

Milton Roberto Furst Crenitte: Conceptualization, Data curation, Investigation, Methodology, Writing (original draft and review and editing).

Leonardo Rabelo de Melo: Conceptualization, data curation, Writing (original draft).

Wilson Jacob-Filho: Conceptualization, data curation, Writing (original draft).

Thiago Junqueira Avelino-Silva: Conceptualization, data curation, Formal Analysis, Methodology, Writing (original draft, review and editing).

## Conflicts of interest

The authors declare no conflicts of interest.

## Appendix Supplementary materials

Image, application 1
