# Supplementary material for: Transforming the invisible into the visible: disparities in the access to health in LGBT+ older people
Source: Clinics (Sao Paulo). 2022 Dec 17;78:100149. doi: 10.1016/j.clinsp.2022.100149 (PMC9791605; doi:10.1016/j.clinsp.2022.100149)
Supplement: Supplementary file 1 [file mmc1.docx]

**CLINICS-D-22-00233_ Supplementary material**

**Supplementary material S1** PCATool-Brasil ‒ First contact: Accessibility (12-items).

| 1. Can you go to your health service on Saturdays or Sundays? |
| --- |
| 2. Can you go to your health service on weekdays evenings until 8-pm? |
| 3. When your health service is open or available. and you get sick. would someone from there see you on the same day? |
| 4. When your health service is closed on Saturday or Sunday, and you get sick. would someone there see you the same day? |
| 5. When your health facility is closed, and you get sick during the night. would someone there see/talk with you that night? |
| 6. Do you have to wait a long time. or speak to many people to schedule appointments at your health service? |
| 7. Is it EASY to schedule an appointment (routine appointment: “check-up”) at this health facility? |
| 8. When the office is closed. is there a phone number you can call when you get sick? |
| 9. When you need to go to your health service do you have to take off from work or school to go? |
| 10. When you arrive at your healthcare facility. do you need to wait more than 30-minutes to consult with the doctor or nurse? |
| 11. Is it DIFFICULT for you to get medical care at your health service when you think it is necessary? |
| 12. When your healthcare facility is open or available. can you get advice over the phone quickly if you need it? |
